# Do you hear the people sing? Comparison of synchronized URL and narrative themes in 2020 and 2023 French protests

**DOI:** 10.3389/fdata.2023.1221744

**Published:** 2023-08-24

**Authors:** Lynnette Hui Xian Ng, Kathleen M. Carley

**Affiliations:** IDeaS, Software and Societal Systems, Carnegie Mellon University, Pittsburgh, PA, United States

**Keywords:** bot detection, social media, synchronization, coordination, network analysis, narrative analysis

## Abstract

**Introduction:**

France has seen two key protests within the term of President Emmanuel Macron: one in 2020 against Islamophobia, and another in 2023 against the pension reform. During these protests, there is much chatter on online social media platforms like Twitter.

**Methods:**

In this study, we aim to analyze the differences between the online chatter of the 2 years through a network-centric view, and in particular the synchrony of users. This study begins by identifying groups of accounts that work together through two methods: temporal synchronicity and narrative similarity. We also apply a bot detection algorithm to identify bots within these networks and analyze the extent of inorganic synchronization within the discourse of these events.

**Results:**

Overall, our findings suggest that the synchrony of users in 2020 on Twitter is much higher than that of 2023, and there are more bot activity in 2020 compared to 2023.

## 1. Introduction

Social media has facilitated citizens to have an active voice in social issues. The ease of use and ubiquity of the Internet and social media platforms has also accelerated the organization of protests. One of the significant protests organized through social media was the 2011 Arab Spring uprisings, where Facebook and Twitter were mainly used to diffuse ideas and information and facilitate inter- and intra-group communication across geographical distances (Wolfsfeld et al., 2013). Another key era of protests is the 2021 ReOpen America protests, a series of collective protests that spread throughout America, which was fueled and amplified by social media (Shugars et al., 2021).

France has experienced recently experienced two protests: one in 2020 and one in 2023. The 2020 protests revolved around the vow from French President Emmanuel Macron to protect the right to caricature the Islamic prophet Muhammad as a cartoon. The vow sparked an outcry from Muslim countries around the world, where tens of thousands of people from Muslim-dominated countries like Pakistan and Lebanon calling for the boycott of France due to the presence of Islamophobia (in Dubai, 2020).

The 2023 protests centered around a pension reform that French President Macron signed into law, which raises the country's retirement age from 62 to 64 years old and extended the number of years of work required for a full pension. The government used Article 49.3 of the Constitution to force the bill through the French Parliament. The pension reform decision sparked a series of civil unrest within the country, leading to widespread street and public transport disruptions, violence in protests, and union-organized strike actions (Jazeera, 2023).

In this study, we further the analysis of social media discourse surrounding protests by tackling the region of France, investigating the synchronization between users on the social media platform. With data gathered from Twitter over two time periods of 2020 and 2023, we analyzed the synchronized dissemination behavior of URLs pointing to external websites as well as the synchronization of users in putting forth narratives. After which, we combine these two ideas of synchronization and identify key clusters of users and describe their portrayal of the events, comparing the information spreading patterns of the two different years.

When comparing both the 2020 and 2023 protest discourse, we ask the following research with respect to the French protests of 2020 and 2023:

1. RQ1: What are the top URLs that are spread among synchronized users?
2. RQ2: What are the top Narratives that are spread among synchronized users?
3. RQ3: Are there differences in the information dissemination between synchronizing bots and humans?
4. RQ4: What are the differences in the user synchrony patterns between the years 2020 and 2023?

Using the results from URL temporal synchronity, narrative similarity throughout the entire discourse, and combining the users that synchronize in both the temporal and network space, we compare the synchronization strategies of users in the 2020 and 2023 French protest discourse to identify patterns of information dissemination. In this study, we make the following contributions:

1. For RQ1, we examine the interplay between external link sources (website, social media links, closed chat links) and Twitter platform. Links shared in 2020 are more news-based, while those shared in 2023 are more geared toward fund-raising and streaming sites.
2. For RQ2, we analyze the prevailing synchronized information dissemination by comparing textual similarities.
3. For RQ3, we identify the presence of organic and inorganic synchronizing users, observing that while bots are more prevalent within the 2023 discourse, they are not as clustered within the 2020 discourse.
4. Overall, for RQ4, We make use of the combination of temporal and narrative synchrony techniques to analyze the synchrony of users within two protests that happen in France. This comparative analysis brings about the differences in the structure of the discourse between both years.

## 2. Related work

Social media provides a straightforward way of disseminating information, presenting experiences, suggesting connections, and effecting changes within a social movement. A string of studies have examined the online discourse surrounding protests. Smith et al. (2015) characterized the different types of social media behavior during a protest. Topping the list are: information dissemination, personal commentary, media surveillance, and criticism of government, media or government supporters. Suárez-Serrato et al. (2016) studied the #YaMeCanse 2014 protest in Mexico, in which 43 teachers disappeared from a rural school, as a gesture of teaching fatigue, while Li et al. (2021) performed temporal and spatial analysis surrounding the 2020 coronavirus lockdown protests to identify public concerns, beliefs, and values. In terms of identifying users that are core protests, Murdock et al. (2023) characterized users who facilitated multi-platform content diffusion during the 2020 US election fraud protest by studying the spread of information through URL posting behaviors across multiple social media platforms.

Within this context, scholarly investigations have also revolved around the use of automated accounts, or bot accounts, to disseminate information online. This includes the spread of both reliable and low-credibility information (Mendoza et al., 2020). In particular, within online protests, bots have been known to be vocal in calling for action: voicing out against the alcoholic beverage act in Indonesia (Danaditya et al., 2022), calling for volunteers to stand up against corruption in Latin America (Savage et al., 2016), and supporting climate change activism (Chen et al., 2021). The examination of the presence and extent of bot accounts provides a differentiation between the inorganic and organic portions of the discourse, in terms of structure and narratives (Tardelli et al., 2022; Ng and Carley, 2023b), which is helpful in determining the potential violence that might result from the cacophony of information dissemination.

The synchronization of users in online discourse These users work together to spread messages faster and they have been discovered to exert more influence within the information cascade (Cinelli et al., 2022). A study of link sharing behavior on Facebook groups and pages between during the 2018 and 2019 Italian elections identified that problematic URLs containing false or misleading information were more likely to be shared by synchronizing users (Giglietto et al., 2020). Within the 2021 US Capitol riots, groups of synchronizing users observed to coordinate the spread of disinformation narratives, such as disputes to the electoral vote count and that Donald Trump still remains as the US president (Ng et al., 2022a). Through the analysis of similar images, two key groups of synchronizing users in the 2019 Hong Kong protests were identified: anti-protest group sharing images with Chinese texts, pro-protest group sharing images with English text.

Several methods have been developed to identify user synchrony on social media. One method of identifying user synchrony is making use of temporal windows, where there is a high level of synchrony between user account interactions such as posting the same URL or retweeting the same person (Giglietto et al., 2020; Weber and Neumann, 2020). Another method applies network science to construct of a weighted user-similarity network, which can be filtered to identify synchronizing communities within the network and how they amplify their influence (Weber and Neumann, 2021). Synchronization can also be identified through the study of similarities between post content, such as similar texts (Ng et al., 2022a) and images (Pacheco et al., 2021). These methods typically work through representing post content as vectors and identifying similar vectors through a distance metric.

In this study, we combined methods for identifying user synchrony on social media to analyze protests that occurred in France.

## 3. Methodology

### 3.1. Data collection

In this work, we analyzed bot networks through two Twitter datasets that depict online discussion on two protests within France—one in 2020 and one in 2023. Both datasets were collected with the Twitter Streaming API V1. The first dataset is the 2020 dataset which contains conversations on the protests related to the French President Macron's crackdown on Islam, with the keywords #frenchprotest2020, #charliehebdo. The second dataset collects conversations related to President Macron's pension reforms in 2023. It was collected using the keywords #frenchprotest2023, #pensionreformprotest. The hashtags for data collection were selected to be broad to capture as much of the conversation as possible. For this study, we retain only the original tweets, i.e., tweets that are not retweets or quote tweets, to have a genuine idea of the original URLs and narratives that are being spread through the conversations. Table 1 shows a summary of the statistics of the datasets.

**Table 1:** Dataset statistics.

	**2020 dataset**	**2023 dataset**
Event	Protests against crackdown on Islam	Protests against pension reforms
Collection timeframe	3–9 Aug 2020	23 March–5 April 2023
Collection keywords	#frenchprotest2020, #charliehebdo	#frenchprotest2023, #pensionreformprotest
Number of tweets	219,188	270,342
Number of users	219,435	124,031
Number of synchronized users (URL)	10,350	120,445
Number of synchronized users (narratives)	82,811	12,256
Number of bots (%)	37,318 (5.80%)	29,196 (23.54%)

Within these data, we only collected publicly available tweets and did not attempt to access accounts that protected their tweets. In this article, we do not reveal the usernames of the analyzed accounts, as some of these accounts are still active.

### 3.2. Bot identification

Many methods have been developed for bot identification. These algorithms use the user's account features such as temporal frequency of tweets (Chavoshi et al., 2016), tweet content (Ng and Carley, 2023a), or even network features (Feng et al., 2021a), to construct bot/human classifiers through the use of supervised machine learning methods, to deep neural network methods (Fazil et al., 2021; Wu et al., 2021) or graph convolutional networks methods (Feng et al., 2021b; Li et al., 2022). In this study, we adopt the BotHunter algorithm (Beskow and Carley, 2018) to classify users into bots and humans. This algorithm constructs supervised random forests, making use of content features, user features, and user network interaction features.

We assign a bot probability score ranging from 0 to 1 for each user within the dataset via the BotHunter classification model. BotHunter is able to work on historical datasets and does not require a real-time pull of the data. As such, we apply this algorithm on the collected and stored data.

The bot probability score indicates a range of likelihood to which the user is likely to be a bot. A score closer to 1 means the user is more likely to be a bot and a score closer to 0 means the user is more likely to be a human. Based on previous studies, we set a score threshold of 0.70, where a user is more likely to be a bot if the score is greater or equal to a probability of 0.70, and a human otherwise (Ng et al., 2022c).

Understanding bot activity throughout the synchronous networks provides an idea of how organic the synchronous URLs and narratives are. Differentiating between the synchronous activities of bot and humans in terms of URLs and narratives spread provides a better understanding of the different focus of the two user classes within the social media ecosystem.

### 3.3. Combined user synchrony network

We construct a Combined User Synchrony Network for each dataset to find synchronizing users across time and narratives. The Combined Synchrony Network combines the information from URL synchrony obtained from users that synchronize in terms of URLs across time, and narrative similarity obtained from users that present similar Narratives across the dataset.

From the raw tweet data, we identify users that synchronize in terms of URLs temporally to form a URL Synchrony Network. We also identify users that synchronize in terms of narratives to create a Narrative Similarity Network. This step returns network graphs where nodes represent users, and a link between two users represents that they synchronize with each other. The number of times they synchronize (i.e., share the same URL or put forth a similar text) is represented by the weight of the link.

Then, we binarize both networks on their own, meaning that instead of counting the number of times two users synchronize, we only take note of which users synchronize with whom within each network. Taking the URL Synchrony Network, for example, if two users, A and B, synchronize with each other in terms of sharing the same URL within their tweet, the weight of the link between A and B will be 1; if two users do not share the same URL as per the URL Synchrony Network, no link will exist between A and B. While this disregards the difference in weights between the connections of users, we are investigating the presence of synchronization between the users and the pattern of synchrony within the event rather than individual user connections.

Finally, we combine both networks into a Combined Synchrony Network, where users that synchronize by URLs or narratives are joined together. In the Combined Synchrony Network, nodes represent users. Two users are connected together if they either synchronized with each other as observed within the URL Synchrony Network, or have presented similar narratives as observed in the Narrative Synchrony Network. This network graph provides a view of the information dissemination patterns for both seasons, and also allows for the extraction of key clusters of users that participate in the conversation.

**Figure 1:**
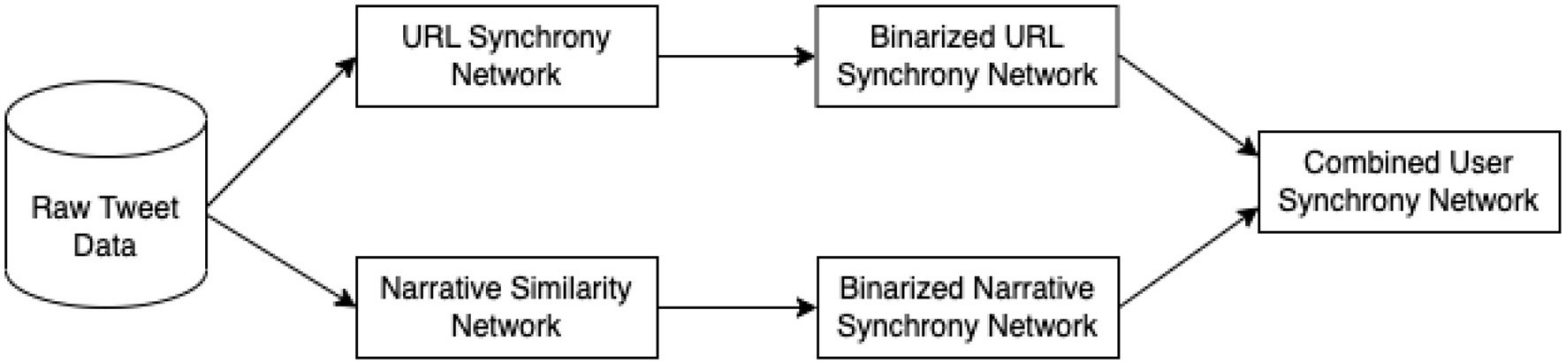
Construction of Combined User Synchrony Network. This network is constructed for the 2020 dataset and the 2023 dataset separately.

Figure 1 shows a flowchart of the construction of the Combined User Synchrony Network. In the next subsections, we elaborate more into constructing the URL and Narrative synchrony networks.

We construct one network for the 2020 dataset and one for the 2023 dataset separately, before performing network analysis and comparison on the French protests of the 2 years.

#### 3.3.1. URL synchrony network

We construct a URL Synchrony Network based on the principles of temporal synchrony. Temporal synchrony occurs when two users frequently post a tweet with the same tweet artifact (i.e., URL, @mention, hashtag) within a short time window of each other. This method has been used in studies that harness the synchrony of users to identify groups that are amplifying influence (Weber and Neumann, 2021), examine coordinated groups in protests (Magelinski et al., 2022), and identify trends of inauthentic information spread on Twitter (Cinelli et al., 2022).

From the raw tweet data, we extract users whose tweets are within 5 min of each other and contain the same expanded URL. We select a 5-min window due to previous studies regarding temporal nuances of highly coordinated communities (Weber and Falzon, 2021; Ng and Carley, 2022; Suresh et al., 2023). A more relaxed timeframe results in a more noisy network, and the users can form chains of synchronization, where user A is joined with user B, who joins with user C, and so forth, resulting in extremely connected clusters in the resultant network. This tight time bound provides more assurance that the users are more likely to be deliberately synchronizing by sharing the same URL within a short period of each other than accidentally synchronizing (Ng and Carley, 2022).

We use URLs as an indicator of synchronization as compared to other tweet artifacts (e.g., hashtag) because URLs show deliberate information referral coordination by pointing to specific pages (Ng and Carley, 2023b). After which, we construct a network graph that represents the URL synchrony. The users that synchronize are represented by the nodes, and an edge joins two synchronizing users. The weight of the edge depicts the number of times the two users synchronize with each other. Then, we perform a thresholding filter (Magelinski et al., 2022) on the graph to weed out noise and keep the core set of synchronizing users. This filter keeps users and links whose weight is above (mean + 1 standard deviation) of the network graph. This URL Synchrony Network graph is later binarized and joined with the Narrative Synchrony Network graph to form a Combined Synchrony Network graph for downstream analysis.

#### 3.3.2. Narrative similarity network

We construct a Narrative Similarity Network graph based on the principles of identification of similar narrative clusters. This method harnesses the fact that similar narratives can be spread across the event discourse but are not necessarily within a short temporal window of each other. Identifying similar narratives has revealed coordinated messaging among Parler users during the 2021 US Capitol Riots (Ng et al., 2022b) and also unveiled coordinated groups behind the 2018–2019 White Helmets disinformation campaign (Pacheco et al., 2020).

For each tweet, we first preprocess it to remove URLs, @mentions, and hashtags, keeping only the core texts of the tweets. Then, we encode the core tweet text into a vector space using the universal sentence encoder (Cer et al., 2018). This encoding provides a vector representation of the words within the core tweet text. The universal sentence encoder is a transformer-based encoder, producing context-aware vector representations of the sentence. A context-aware vector representation is useful in identifying narrative synchrony by spotting sentences that are similar in idea but are not necessarily identical word for word.

Next, we construct a tweet-to-tweet graph of similar texts. We perform an all-texts comparison throughout the entire set of tweet texts, comparing the encoded text vectors via a cosine similarity measure. The cosine similarity metric measures how close two text vectors are. The metric ranges from a scale of 0–1, where 1 means the two vectors are identical, and 0 means the two vectors are non-identical. In the tweet-to-tweet graph, tweets are represented by nodes, and links between the nodes are weighted by the cosine similarity between the two tweet vectors. To keep only narratives that are extremely similar to each other, we only retain nodes and links where the cosine similarity score is above the 0.70 threshold. Past studies that compare textual similarity in tweet texts use thresholds ranging from 0.60 to 0.80 (Ozdikis et al., 2012; Iyer et al., 2017; Ng et al., 2022a; Ravi and Kulkarni, 2022), and as such, we use an average of these thresholds (0.70).

After which, we fold the tweet-to-tweet network graph to construct a Narrative Similarity Network, which represents which users are more similar to each other in terms of the tweets they post. For each tweet, we identified the corresponding user that authored the tweet. For each tweet-tweet pair in the tweet-to-tweet network graph, we convert it into a user-user pair, matching the users that authored the tweets. Two users would have a link between them if they have a link within the tweet-to-tweet graph. The weights of the user-user link represent the number of times the tweets of the two authors are connected together in the original tweet-to-tweet network graph.

In this Narrative Similarity Network, we first map each tweet to its corresponding user. Users affiliated with similar tweets are deemed synchronizing users, and are joined together by an edge. The weight of the edge depicts the number of times the two users synchronize with each other, or have similar narratives within their tweets. Then, we perform a thresholding filter (Ng et al., 2022a) on the graph to weed out noise and keep the core set of synchronizing users. This filter keeps users and links whose weight is above (mean + 1 standard deviation) of the network graph.

This Narrative Similarity Network graph is later binarized and joined with the URL Synchrony Network graph to form a Combined User Synchrony Network graph for downstream analysis.

### 3.4. Comparative analysis of top synchronous URLs and narratives

With the construction of URL Synchrony Network and Narrative Similarity Network, we parsed the top URLs and Narratives that are constantly shared between users through the dataset. For URLs, we categorize the URLs into the base URLs, which is the root part of the URL address. This categorization reflects the key sites that are being referred to within the discourse. This analysis provides an idea of the most frequently used external websites and topic themes that are used within the online discourse during the event.

We further do this parsing by bot/human user classes, which aids in differentiating the types of URLs and Narratives that are organically and inorganically spread.

### 3.5. Comparative analysis of combined synchronous networks of 2020 and 2023

We analyzed how the Combined User Synchrony Networks of the two French events are similar or dissimilar. We first perform Louvain clustering on the networks to identify key clusters. This technique aids in discovering the internal groupings of users within the networks using a community detection method which detects clusters with high connectivity through local optimization and aggregation (Yoshida et al., 2021). Following this, we extract the narratives and URLs that the users in each Louvain cluster disseminate and manually interpret them. We also annotate and analyze the presence of bots within each set of networks, profiling the type of conversations for the events.

## 4. Results

France has seen two major protests: one in 2020 against the crackdown of Islam, and another in 2023 against the pension reforms. This section presents the results of users synchronizing via URLs and Narrative themes during these two protests.

### 4.1. Comparison of top synchronous URLs

Users synchronize in terms of their presentation of the same URLs within a short 5-min time window. We break down these URLs into the base domains, which represent the umbrella under which a few sites reside under.

Table 2 shows the differences between the 2020 and 2023 datasets in terms of the base URLs that are frequently shared among users. In 2020, the URLs shared are specific links to news sites, and there are links to Indonesian sites too. This reflects the nature of the event, where it is centered around the Muslim community, of which Indonesia has a huge Muslim community. URL links from 2023 point to fund-raising sites (e.g., “ko-fi.com”), groups on other social media (e.g., Telegram and WhatsApp), and streaming sites (e.g. “twitch.tv,” “kick.com”). These show a change in the trends of the URLs that are being shared: from news sites to fund-raising sites.

**Table 2:** Top base domains of URLs within the URL synchrony network.

	**2020 dataset**		**2023 dataset**	
	**Base URL**	**Count**	**Base URL**	**Count**
Bots	twitter.com	5,374	twitter.com	1190
	bit.ly	3,524	ko-fi.com	348
	dlvr.it	1,894	t.me	172
	republika.co.id	1,233	youtube.com	132
	news.idtoday.co	1,024	amazon.com	27
Humans	twitter.com	17,057	twitter.com	3,216
	dlvr.it	2,269	ko-fi.com	483
	youtube.com	1,970	youtube.com	243
	news.detik.com	594	twitch.tv	198
	instagram.com	553	kick.com	66

Additionally, we also see a difference in the base URLs shared by bots and humans. In the 2020 dataset, there is a small difference, as both types of users share links that point to news sites. However, humans also share links to other social media sites like YouTube and Instagram, sharing information in terms of videos and images. In the 2023 dataset, bots generally share links to affiliate social media and purchase sites like Telegram, KoFi, Amazon, and YouTube, while humans share links to streaming sites like Kick and Twitch.

### 4.2. Comparison of top similar narratives

Users also synchronize in terms of the Narratives they put forth in their tweets, expressing their opinion or ideologies. We identify the top similar Narratives that circulate within the discourse of each of the events and manually interpret them. Table 3 show the top similar Narratives that are disseminated by bot accounts and Table 4 tabulates the top similar Narratives that are disseminated by human accounts.

**Table 3:** Top narratives within narrative similarity network for bots.

	**2020 dataset**		**2023 dataset**	
	**Narrative**	**Example tweets**	**Narrative**	**Example tweets**
Bots	Criticism of French President Macron	#ShameOnYourMacron France is promoting Islamophobia. Islamophobia is terrorism. Macron listen!!! You're activities are a threat to world peace bcz you have crossed the red line that no Muslim can tolerate [...]	Breakdown of Democracy	What the breakdown of democracy looks like........ #macron #ResignMacron This is not democracy it's tyranny against unarmed citizens. #Macron #France #Tyranny
	Thoughts on cartoon	[...] Macron: France will not give up our cartoons [...] Strongly condemn French pres Macron's acts who allowed display of blasphemous cartoons, hurting sentiments of billions [...]	Linking to external broadcast sites	[...] We broadcast on telegram: Link https://t.co/De5rAk4ah4. The situation is degenerating Bordeaux Marseille Lyon Paris and everywhere in France tonight #Greve23Mars #manif23mars #macron #Francia #Frankreich Strike Pension Reform Protesters from all over France call to converge on #Paris Tomorrow Telegram Groups [...]
	Quotes from the Prophet Muhammad	Prophet Muhammad said “All the sons of Adam are sinners but the best of sinners are those who repent often” [...] Prophet Muhammad: Charity does not in any way decrease the wealth and the servant who forgives Allah [...]	References to Charles III of United Kingdom	On the contrary, #CharlesIII must come and the whole visit must be a humiliating fiasco for #Macron [...] Since he no longer receives the King of England #CharlesIII #Macron can receive the intersyndicale now

Example tweets are kept in the raw form as per the original writing. User names and @mentions are removed to preserve user privacy. Where there are [...] it suggests the removal of user names, @mentions, or URLs for brevity.

**Table 4:** Top narratives within narrative similarity network for humans. Example tweets are kept in the raw form as per the original writing.

	**2020 dataset**		**2023 dataset**	
	**Narrative**	**Example tweets**	**Narrative**	**Example tweets**
Humans	Calls to boycott French products	We can sacrifice anything for the dignity of Our Holy Prophet Muhammad ... #boycottfrenchproduct #FranceShameOnYou [...] We love our prophet very much. If anyone insults our Prophet we will not remain silent. #boycottfrenchproduct [...]	Police activities/ violence	[...]A tractor loads the water cannon of the police they don't laugh the Bretons!! French police are better equipped against unarmed French citizens than [the French army] against the Taliban
	Thoughts on cartoon	Charlie Hebdo had always been Anti Religion. They mock every religion. These Cartoons didn't lead to any beheading or killing. [...] [...] Muslims believe that any depiction of the prophet Muhammad PBUH is blasphemous. Stop publication of blasphemous cartoons	Targeting the French President	French President #Macron wants to impose his pension reform on the National Assembly via 49.3 The president of the UPR calls on the deputies to launch an impeachment procedure against Emmanuel #Macron [...]I knew there was going to be trouble when #Macron was “elected” [...]
	References to the Quran	Verse taken from Quran (At-Tawbah 9:61) those who distress the Prophet, saying: “He is all ears.” #BoycottFrance #boycottfranceproducts #MohammadTheProphetOfPeace [...] Islam is a religion of mercy; Hazrat Muhammad (saw) the Prophet of mercy and Holy Quran the book of mercy. [...]	References to the musical Les Miserables	Hey #Macron how's your ban on protests going? Do you hear the people sing? #Paris [...]

User names and @mentions are removed to preserve user privacy. Where there are [...] it suggests the removal of user names, @mentions, or URLs for brevity.

In 2020, the top similar Narratives disseminated by both bot and human accounts are roughly similar: thoughts on the cartoon that sparked the protests and quotes from the Islamic Prophet Muhammad or Islamic religious text, the Quran. However, while bots made effort to criticize the French president, humans called for the boycott of French products, signaling their differences in focus.

For the 2023 protest dataset, bots and humans have different narratives and agendas. Bots talk about the breakdown of democracy with the use of the constitution to pass the pension reform law and made links to external broadcast sites. Bot accounts also made reference to the visit of King Charles III of the United Kingdom to the French president. In contrast, human accounts reference the fictional musical Les Miserables about reformation. The accounts also target visible police activities and violence and criticize the French president.

### 4.3. Comparison of combined user synchrony networks

From the synchrony of URLs and narratives between users, we combine the data to construct a Combined User Synchrony Network for each dataset. This Combined User Synchrony Network graph allows us to visualize the users that synchronize using both URL and Narratives, allowing us to dig into the patterns of the information dissemination within the discourse of each event.

**Figure 2:**
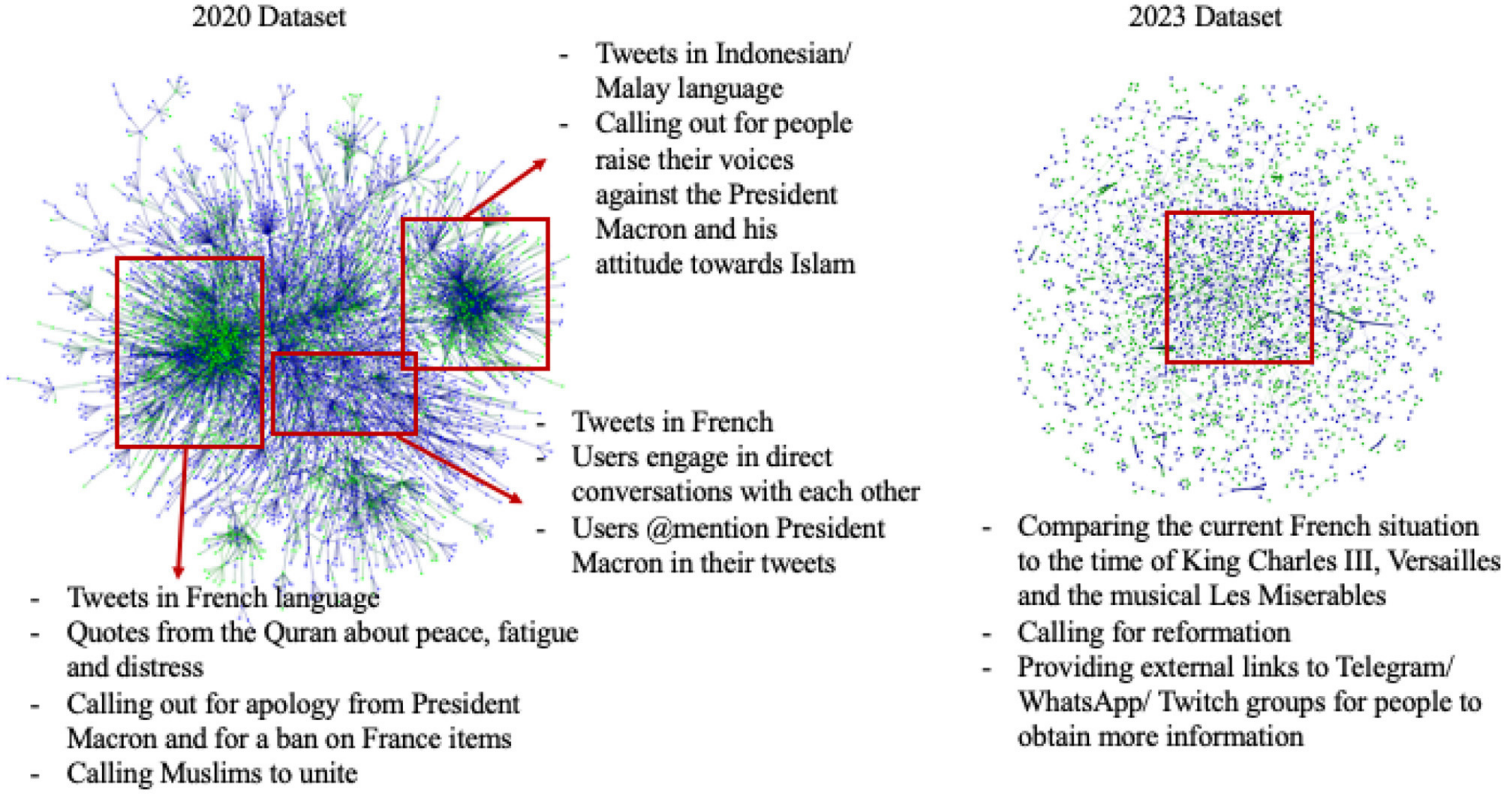
Combined User Synchrony Networks of 2020 and 2023 datasets respectively. Green nodes are bot users and blue nodes are human users. The key clusters are identified through Louvain cluster analysis.

Figure 2 shows the network graphs of the Combined Synchronous Networks. The network graphs of the 2020 and 2023 datasets differ in terms of the structure of clusters among the users. Through Louvain clustering, the 2020 dataset shows three prominent clusters: tweets calling out for people to raise their voices against French President Macron and his attitude toward Islam; tweets engaging in direct conversations with each other; and tweets that quote from the Quaran, the religious text of Islam. However, the 2023 dataset does not show clear clusters of users. In general, the users tweet three main storylines: comparing the current French situation of pension reforms to historical eras such as that of King Charles III and the Versailles era, and even of the musical Les Miserables; call for reformation; and provide external links to chat-based social media such as Telegram and WhatsApp for people to obtain more information.

While there is a higher percentage of bots that are present in the 2023 discourse, the 2020 discourse shows more aggregation of bot users within clusters. The 2023 network graph presents the bot and human users in a more distributed fashion rather than a singly clustered one.

In general, the 2020 dataset shows clear and segregated clusters, while the 2023 dataset does not present any clear core groups. The difference in the network grouping patterns reflects the shift in the social media discourse between years: from prominent networks of synchronizing users to a hub-and-spoke model, which is a central core group with peripheral clusters of users. Table 5 shows the comparison of network statistics between both years. The 2020 dataset presents a higher density and echo chamberness, suggesting users cluster together in groups that reinforce similar beliefs. The echo chamber measure is derived from the ratio of the in-group links to the out-group links. The 2023 dataset presents more fragmentation and more cliques than the 2020 dataset, which is consistent with a hub-and-spoke model. The network pattern of the 2020 dataset presents a higher transitivity or clustering coefficient compared to the 2023 dataset, which means that the network contains more communities that are densely connected internally, as depicted in our network graph. As a result of this network structure, the users in the 2023 dataset have a higher betweenness centrality, meaning they are better able to pass information across the network through their connections. The 2023 network also have a higher closeness centrality, which means the users have shorter distances to all other users as compared to the users in the 2020 dataset, making information spread extremely efficient.

**Table 5:** Network statistics of 2020 and 2023 datasets.

**Network statistic**	**2020 dataset**	**2023 dataset**
Density	1.24E-3	7.74E-4
Clique count	266	2,156
Echo chamberness	0.107	0.092
Avg betweeness	0.106	0.119
Avg closeness	3.11E-4	0.163
Avg transitivity	0.189	0.425
Fragmentation	0	0.714

## 5. Discussion

### 5.1. Changes in URL synchrony pattern

The profiles of top synchronous URLs depicts how online activism have shifted from news dissemination to fundraising campaigns for the offline protests. In 2020, URLs were focused on disseminating up-to-date news and opinions of key personalities to supporters online. In 2023, the synchronizing URLs shifted focus to coordinating funding and logistics that can be used in the offline protest. Such coordination efforts have previously been seen in the 2012 Syrian protest, where an online campaigning group raised money with an online model and acted as an offline logistical supply chain route to support the offline protesters (Pilkington, 2012). Another key shift of the 2023 synchronizing URLs is the use of streaming sites for situational information about the protest rather than using news sites like in 2020. Streaming sites provide real-time situational information, while information from the news sites is delayed by the news cycle.

### 5.2. Differences in narrative similarity pattern

The profiles observed in Narrative similarity patterns between bot and human user types differ between 2020 and 2023. In 2020, the two user classes put forth the same type of narratives. In 2023, the bot users are activated to post links to external broadcast sites, a narrative that automation can be harnessed to do. Bot users chant about the breakdown of democracy within the French region, consistent with previous observations that bot users echo calls for action (Savage et al., 2016; Danaditya et al., 2022). Human users provide observations of police activity and violence. This reflects the usage of bot automation to disseminate information that can be pre-canned, i.e., thoughts on the loss of democracy; but human users provide observational information from ground zero, i.e., location of the police, their weapons, and their actions.

### 5.3. Combining URL and narrative synchronization

By combining URL synchronization network and Narrative Similarity Network into a Combined User Synchrony Network, we employ a network-centric method to observe the differences in the information dissemination patterns of 2020 and 2023.

The 2020 network presents as a highly dense network, with easily identifiable clusters. A dense network structure with distinct clusters signifies separate groups of people that synchronize with each other, overlapping in the temporal space in terms of the same URLs and narratives. Each group has a different set of narratives that they are springing to life. Similarly, the different user types are aggregated by clusters, where bot and human users are generally amassed in separate clusters.

The 2023 network, however, shows a different information dissemination pattern: it mimics a hub-and-spoke model. The core of the network contains more users, after which the network disperses out, indicating that there is a core group of users that push key narratives which then propagate to the peripherals of the network. This is also consistent with the top synchronous URLs being fundraising sites, thereby suggesting the central group of users within the network graph act as coordinating hub and supply users. With such a network pattern, the different user types are also more dispersed, and bots and humans do synchronize with each other.

A hub and spoke network model that is observed in the 2023 protests allows for increased distribution capacity during peak periods, facilitating fast and efficient information flow, which is ideal in a protest situation. This model is commonly used in logistics routing or airline route planning for optimization of goods and services flow (Cook and Goodwin, 2008). Similarly, a hub and spoke model for information dissemination provides a more optimized way of spreading messages within the social media network. Such a model also has a hierarchical component to it: the hub is the core in which most messages originate from, and the spokes are the peripheral clusters which receive and further disseminate the information. In contrast, the model of the 2020 protests in which there is a dense network with distinct groups of users results in echo-chambers, where each group of users propagate their own ideology mostly within the group and have minimal interaction with users outside the group. While there is a lot of information sharing within the group, there is little information sharing across groups. Overall, a message does not pass through the network as efficiently as a hub-and-spoke model due to the clique formation of the users. This model is thus not as efficient in reaching large groups of people for the purposes of organizing collective action online.

In this paper, we analyzed only tweets in the English language, which has an impact in interpreting the users that synchronize. By selecting only for tweets in the English language, we are selecting for users that are targeting their messages at the general population, and not only those who read French. With this selection of users in our data, the voice that is being represented comes from all around the world. Table 6 shows the top five countries that are indicated in the data. That is, the countries that users have chosen to reveal on Twitter as their primary location.

**Table 6:** Most common countries where users are located.

**2020 dataset**		**2023 dataset**	
Pakistan	11.52%	France	58.50%
Turkey	7.23%	Mexico	0.17%
France	5.35%	Venezuela	0.17%
Malaysia	3.52%	England	0.15%
Indonesia	2.50%	United States	0.12%
Country not indicated	18.39%	Country not indicated	28.89%

With the different events, different voices are being represented in the event. In the 2020 dataset, users come from countries that have a largely Muslim population: Pakistan, Turkey, Malaysia, Indonesia. In 2023, half of the users presented their location as being from France. This distribution of location among the users also plays out in the interaction of users between the years, and how the people react to the different events. The 2020 protests were targeted toward a more international issue of hate toward the Muslim and/or Islamic community, in which Muslim communities and leaders around the world made statements about their position against the decision of the French President (Ariffin and Hussain, 2021). In contrast, the 2023 protests were targeted toward a more domestic issue, concentrated around stressing solidarity and the role of pension systems (Väänänen and Liukko, 2023).

Overall, our observations show that the online discourse on French protest has evolved from a clustered response to more dispersed response from 2020 to 2023. The clustered response has segregated narratives and user groups that can be extracted and investigated. The 2023 network shows that user groups are becoming more assimilated and are infusing narratives in a more centralized fashion. This manner of information dissemination could be a new form of protest organization, where information is first consolidated by a core group of users and dispersed to the peripherals, thereby reaching as large a number of people with the same messaging. In addition, the peripheral participants can be critical to increasing the reach of protests messages, and success in spreading the ideology of the core users is due to the success in maximizing the number of online users exposed to the messages, in which the peripheral users play a critical role (Barberá et al., 2015).

### 5.4. Limitations and future work

Several limitations nuance the generalizability of the findings from this work. The Twitter API retrieval technique used returns only a 1% sample of Tweets. Since our datasets are curated using selected keywords within a certain timeframe, it shows only a subset of the full interactions present on the social media platform. Thus, there may be synchronizing users that are not captured in the datasets.

Second, the data is collected and analyzed in the English language, which is not fully representative of all the French narratives, nor is it a random sample of all users that are expressing their opinions about the event. Future work involves collecting data across multiple languages using a wider range of keywords. It also involves understanding narratives across different languages, such as making use of language-agnostic text vectorization methods so texts can across multiple languages can be compared, especially so in the native language in which the event had occurred.

Lastly, the bot identification algorithm used was trained on a series of curated datasets ranging from political to financial to spam bots. While the large training dataset does lend weight to its generalizability, the bot algorithm may not necessarily be trained on a protest dataset and might not be equally as effective in identifying bots within a protest event. However, we overcome this problem by identifying groups of users through their synchronous actions, in terms of URLs and Narrative synchronity to make sense of the discourse during each event.

## 6. Conclusion

Synchronization of users in social media can provide a glimpse of the user accounts working together to push certain narratives in the online discourse. Narratives and ideals disseminated in the online space can lead to offline real-world actions, as portrayed in the two French protests that are studied in this paper. The extraction of synchronized URLs and narratives provides clues to conversational trends, and the combination of synchronous networks allow the examination of patterns of information dissemination.

In this work, we compared the social media discourse across two protests that occurred in France. Through the use of analyzing URL synchrony, we are able to understand the key external links that are referred to frequently by the users on Twitter. The synchrony of Narratives within the discourse provides glimpses of the information dissemination across the platform. The Combined Synchrony Network provides a network-centric view to examine the synchronization pattern within the discourse.

In this study, we identify that the URLs disseminated in 2020 and 2023 differ: in 2020, URLs referred to webpages that provided news and information, while in 2023, URLs referred to webpages for crowdfunding and where users can purchase items. In terms of narrative synchronization, bots and humans presented more similar narratives in 2020 while in 2023, the narratives diverged. Lastly, we observe that in terms of user synchrony, the 2020 dataset presents more segregated clusters and bot activity, while the 2023 dataset presents a hub-and-spoke model where there are no clear separate groups of users but rather a central core group of synchronous users and peripheral ties.

We hope the techniques presented in this work can be further used to analyze and compare user synchrony between events and streamline online discourse for deeper investigation.

## Data availability statement

The data analyzed in this study is subject to the following licenses/restrictions: The datasets come from Twitter. The datasets can be made available in correspondence to the Terms and Conditions of Twitter; i.e., only certain part of the data to be available and the recipient needs to rehydrate the data. Requests to access these datasets should be directed to LN, lynnetteng@cmu.edu.

## Ethics statement

Ethical approval was not required for the study involving human data in accordance with the local legislation and institutional requirements. The datasets were collected using Twitter Streaming API V1.

## Author contributions

LN contributed in conceptualization, data collection and analysis, and writing of the manuscript. KC contributed on conceptualization and data collection. All authors approved the manuscript.

## References

[B1] Ariffin N. I. Hussain F. (2021). The 2020 France attacks: a framing analysis of UK and US newspapers. Int. J. Modern Trends Soc. Sci. 4, 133–146. 10.35631/IJMTSS.4150012

[B2] Barberá P. Wang N. Bonneau R. Jost J. T. Nagler J. Tucker J.. (2015). The critical periphery in the growth of social protests. PLoS ONE 10:e0143611. 10.1371/journal.pone.0143611 26618352 PMC4664236

[B3] Beskow D. M. Carley K. M. (2018). “Bot-hunter: a tiered approach to detecting & characterizing automated activity on twitter,” in Conference Paper. SBP-BRiMS: International Conference on Social Computing, Behavioral-Cultural Modeling and Prediction and Behavior Representation in Modeling and Simulation, Vol. 3 (Cham: Springer).

[B4] Cer D. Yang Y. Kong S.-Y. Hua N. Limtiaco N. John R. S.. (2018). Universal sentence encoder. arXiv preprint arXiv:1803.11175. 10.18653/v1/D18-2029

[B5] Chavoshi N. Hamooni H. Mueen A. (2016). “Debot: Twitter bot detection via warped correlation,” in 2016 IEEE 16th International Conference on Data Mining (ICDM) (Barcelona: IEEE), 817–822. 10.1109/ICDM.2016.0096

[B6] Chen C.-F. Shi W. Yang J. Fu H.-H. (2021). Social bots' role in climate change discussion on twitter: measuring standpoints, topics, and interaction strategies. Adv. Clim. Change Res. 12, 913–923. 10.1016/j.accre.2021.09.011

[B7] Cinelli M. Cresci S. Quattrociocchi W. Tesconi M. Zola P. (2022). Coordinated inauthentic behavior and information spreading on twitter. Decis. Support Syst. 160:113819. 10.1016/j.dss.2022.11381935301344

[B8] Cook G. N. Goodwin J. (2008). Airline networks: a comparison of hub-and-spoke and point-to-point systems. J. Aviat. Aerospace Educ. Res. 17:1. 10.15394/jaaer.2008.1443

[B9] Danaditya A. Ng L. H. X. Carley K. M. (2022). From curious hashtags to polarized effect: profiling coordinated actions in Indonesian twitter discourse. Soc. Netw. Anal. Mining 12:105. 10.1007/s13278-022-00936-2

[B10] Fazil M. Sah A. K. Abulaish M. (2021). Deepsbd: a deep neural network model with attention mechanism for socialbot detection. IEEE Trans. Inform. Forens. Secur. 16, 4211–4223. 10.1109/TIFS.2021.3102498

[B11] Feng S. Wan H. Wang N. Li J. Luo M. (2021a). “Twibot-20: a comprehensive twitter bot detection benchmark,” in Proceedings of the 30th ACM International Conference on Information & *Knowledge Management* (New York, NY: Association for Computing Machinery), 4485–4494. 10.1145/3459637.3482019

[B12] Feng S. Wan H. Wang N. Luo M. (2021b). “BOTRGCN: Twitter bot detection with relational graph convolutional networks,” in Proceedings of the 2021 IEEE/ACM International Conference on Advances in Social Networks Analysis and Mining (New York, NY: Association for Computing Machinery), 236–239. 10.1145/3487351.3488336

[B13] Giglietto F. Righetti N. Rossi L. Marino G. (2020). It takes a village to manipulate the media: coordinated link sharing behavior during 2018 and 2019 Italian elections. Inform. Commun. Soc. 23, 867–891. 10.1080/1369118X.2020.1739732

[B14] in Dubai A. P. (2020). Anti-France Protests Draw Tens of Thousands Across Muslim World. theguardian.com. Available online at: https://www.theguardian.com/world/2020/oct/30/anti-france-protests-draws-tens-of-thousands-across-muslim-world [accessed May 02, 2023).

[B15] Iyer R. Wong J. Tavanapong W. Peterson D. A. (2017). “Identifying policy agenda sub-topics in political tweets based on community detection,” in Proceedings of the 2017 IEEE/ACM International Conference on Advances in Social Networks Analysis and Mining 2017 (New York, NY: Association for Computing Machinery), 698–705. 10.1145/3110025.3116208

[B16] Jazeera A. (2023). Macron signs France pension reform into law despite protests. Al Jazeera. Available online at: https://www.aljazeera.com/news/2023/4/15/macron-signs-france-pension-reform-into-law-despite-protests

[B17] Li L. Erfani A. Wang Y. Cui Q. (2021). Anatomy into the battle of supporting or opposing reopening amid the COVID-19 pandemic on twitter: a temporal and spatial analysis. PLoS ONE 16:e0254359. 10.1371/journal.pone.0254359 34255783 PMC8277023

[B18] Li S. Zhao C. Li Q. Huang J. Zhao D. Zhu P. (2022). BotFinder: a novel framework for social bots detection in online social networks based on graph embedding and community detection. World Wide Web 1–17. 10.21203/rs.3.rs-1871702/v1

[B19] Magelinski T. Ng L. Carley K. (2022). A synchronized action framework for detection of coordination on social media. J. Online Trust Saf . 1. 10.54501/jots.v1i2.30

[B20] Mendoza M. Tesconi M. Cresci S. (2020). Bots in social and interaction networks: detection and impact estimation. ACM Trans. Inform. Syst. 39, 1–32. 10.1145/3419369

[B21] Murdock I. Carley K. M. Yaúgan O. (2023). Identifying cross-platform user relationships in 2020 us election fraud and protest discussions. Online Soc. Netw. Media 33:100245. 10.1016/j.osnem.2023.100245

[B22] Ng L. H. X. Carley K. M. (2022). “Online coordination: methods and comparative case studies of coordinated groups across four events in the United States,” in 14th ACM Web Science Conference 2022 (New York, NY: Association for Computing Machinery), 12–21. 10.1145/3501247.3531542

[B23] Ng L. H. X. Carley K. M. (2023a). “Botbuster: multi-platform bot detection using a mixture of experts,” in Proceedings of the International AAAI Conference on Web and Social Media, Vol. 17 (PKP Publishing Services), 686–697. 10.1609/icwsm.v17i1.22179

[B24] Ng L. H. X. Carley K. M. (2023b). A combined synchronization index for evaluating collective action social media. Appl. Netw. Sci. 8:1. 10.1007/s41109-022-00526-3 36620080 PMC9809510

[B25] Ng L. H. X. Cruickshank I. J. Carley K. M. (2022a). Coordinating narratives framework for cross-platform analysis in the 2021 us capitol riots. Comput. Math. Organ. Theory 1–17. 10.1007/s10588-022-09371-2 36440374 PMC9676754

[B26] Ng L. H. X. Cruickshank I. J. Carley K. M. (2022b). Cross-platform information spread during the January 6th capitol riots. Soc. Netw. Anal. Mining 12:133. 10.1007/s13278-022-00937-1 36105923 PMC9461432

[B27] Ng L. H. X. Robertson D. C. Carley K. M. (2022c). Stabilizing a supervised bot detection algorithm: how much data is needed for consistent predictions? Online Soc. Netw. Media 28:100198. 10.1016/j.osnem.2022.100198

[B28] Ozdikis O. Senkul P. Oguztuzun H. (2012). “Semantic expansion of tweet contents for enhanced event detection in twitter,” in 2012 IEEE/ACM International Conference on Advances in Social Networks Analysis and Mining (IEEE), 20–24. 10.1109/ASONAM.2012.14

[B29] Pacheco D. Flammini A. Menczer F. (2020). “Unveiling coordinated groups behind white helmets disinformation,” in Companion Proceedings of the Web Conference 2020, WWW '20 (New York, NY: Association for Computing Machinery), 611–616. 10.1145/3366424.3385775

[B30] Pacheco D. Hui P.-M. Torres-Lugo C. Truong B. T. Flammini A. Menczer F. (2021). “Uncovering coordinated networks on social media: methods and case studies,” in Proceedings of the International AAAI Conference on Web and Social Media, Vol. 15 (AAAI Press), 455–466. 10.1609/icwsm.v15i1.18075

[B31] Pilkington E. (2012). Avaaz Faces Questions Over Role at Centre of Syrian Protest Movement. The Guardian, 2.

[B32] Ravi J. Kulkarni S. (2022). “Finding spatial-textual clusters in COVID tweets,” in 2022 International Conference on Advances in Computing, Communication and Applied Informatics (ACCAI), 1–6. 10.1109/ACCAI53970.2022.9752658

[B33] Savage S. Monroy-Hernandez A. Höllerer T. (2016). “Botivist: calling volunteers to action using online bots,” in Proceedings of the 19th ACM Conference on Computer-Supported Cooperative Work & *Social Computing*, 813–822. 10.1145/2818048.2819985

[B34] Shugars S. Gitomer A. McCabe S. Gallagher R. J. Joseph K. Grinberg N.. (2021). Pandemics, protests, and publics: demographic activity and engagement on twitter in 2020. J. Quant. Descript. 1. 10.51685/jqd.2021.002

[B35] Smith B. G. Men R. L. Al-Sinan R. (2015). Tweeting taksim communication power and social media advocacy in the taksim square protests. Comput. Hum. Behav. 50, 499–507. 10.1016/j.chb.2015.04.012

[B36] Suárez-Serrato P. Roberts M. E. Davis C. Menczer F. (2016). “On the influence of social bots in online protests: preliminary findings of a Mexican case study,” in Social Informatics: 8th International Conference, SocInfo 2016 (Bellevue, WA: Springer), 269–278. 10.1007/978-3-319-47874-6_19

[B37] Suresh V. P. Nogara G. Cardoso F. Cresci S. Giordano S. Luceri L. (2023). Tracking fringe and coordinated activity on twitter leading up to the US capitol attack. arXiv preprint arXiv:2302.04450. 10.48550/arXiv.2302.04450

[B38] Tardelli S. Avvenuti M. Tesconi M. Cresci S. (2022). Detecting inorganic financial campaigns on twitter. Inform. Syst. 103:101769. 10.1016/j.is.2021.101769

[B39] Väänänen N. Liukko J. (2023). Justifying a financially and socially sustainable pension reform: a comparative study of Finland and France. Int. J. Sociol. Soc. Policy 43, 507–520. 10.1108/IJSSP-04-2022-0091

[B40] Weber D. Falzon L. (2021). Temporal nuances of coordination network semantics. arXiv preprint arXiv:2107.02588. 10.48550/arXiv.2107.0258819642889

[B41] Weber D. Neumann F. (2020). “Who's in the gang? Revealing coordinating communities in social media,” in 2020 IEEE/ACM International Conference on Advances in Social Networks Analysis and Mining (ASONAM) (Austria: Springer), 89–93. 10.1109/ASONAM49781.2020.9381418

[B42] Weber D. Neumann F. (2021). Amplifying influence through coordinated behaviour in social networks. Soc. Netw. Anal. Mining 11:111. 10.1007/s13278-021-00815-2 34745379 PMC8557266

[B43] Wolfsfeld G. Segev E. Sheafer T. (2013). Social media and the Arab spring: politics comes first. Int. J. Press 18, 115–137. 10.1177/1940161212471716

[B44] Wu Y. Fang Y. Shang S. Jin J. Wei L. Wang H. (2021). A novel framework for detecting social bots with deep neural networks and active learning. Knowl. Based Syst. 211:106525. 10.1016/j.knosys.2020.106525

[B45] Yoshida M. Sakaki T. Kobayashi T. Toriumi F. (2021). Japanese conservative messages propagate to moderate users better than their liberal counterparts on twitter. Sci. Rep. 11:19224. 10.1038/s41598-021-98349-2 34602612 PMC8488035

