# Corrigendum: Do you hear the people sing? Comparison of synchronized URL and narrative themes in 2020 and 2023 French protests

**DOI:** 10.3389/fdata.2023.1343108

**Published:** 2023-12-12

**Authors:** Lynnette Hui Xian Ng, Kathleen M. Carley

**Affiliations:** IDeaS, Software and Societal Systems, Carnegie Mellon University, Pittsburgh, PA, United States

**Keywords:** bot detection, social media, synchronization, coordination, network analysis, narrative analysis

In the published article, there was an error in Table 1 as published. For the row of Number of Synchronized Users (URL), the value should be 10,350 and not 643,956. There was also a mistake in the caption, it should be “Dataset Statistics” and not “Datasets statistics.” The corrected Table 1 and its caption appear below.

**Table 1:** Dataset statistics.

	**2020 dataset**	**2023 dataset**
Event	Protests against crackdown on Islam	Protests against pension reforms
Collection timeframe	3–9 Aug 2020	23 March – 5 April 2023
Collection keywords	#frenchprotest2020, #charliehebdo	#frenchprotest2023, #pensionreformprotest
Number of tweets	219,188	270,342
Number of users	219,435	124,031
Number of synchronized users (URL)	10,350	120,445
Number of synchronized users (narratives)	82,811	12,256
Number of bots (%)	37,318 (5.80%)	29,196 (23.54%)

The authors apologize for this error and state that this does not change the scientific conclusions of the article in any way. The original article has been updated.

